# Native Species Facing Climate Changes: Response of Calafate Berries to Low Temperature and UV Radiation

**DOI:** 10.3390/foods10010196

**Published:** 2021-01-19

**Authors:** María Eugenia Romero-Román, Mauricio Schoebitz, Richard M. Bastías, Pablo S. Fernández, Cristina García-Viguera, María Dolores López-Belchi

**Affiliations:** 1 Departamento de Producción Vegetal, Facultad de Agronomía, Universidad de Concepción, Campus Chillán 3780000, Chile; mariaeugeromero@udec.cl (M.E.R.-R.); ribastias@udec.cl (R.M.B.); 2 Food Engineering and Agricultural Equipment Department, Universidad Politécnica de Cartagena, 30203 Cartagena, Spain; pablo.fernandez@upct.es; 3 Departamento de Suelos y Recursos Naturales, Facultad de Agronomía, Universidad de Concepción, Concepción 4030000, Chile; mschoebitz@udec.cl; 4 Phytochemistry Labaratoty Research Group on Quality, Safety and Bioactivity of Plant Foods, Department of Food Science and Technology, CEBAS (CSIC), 30100 Campus University Espinardo, 30100 Murcia, Spain; 5 Unidad Asociada de Calidad y Evaluación de Riesgos de Alimentos, CEBAS (CSIC)—UPCT, 30100 Murcia, Spain

**Keywords:** native Chilean berry, antioxidant activity, UV radiation, temperature influence, PCA, food security, climate change

## Abstract

Calafate (*Berberis microphylla* G. Forst) is a wild bush plant widely distributed in the south of Argentina and Chile. Their blue colored fruits present particular flavor and health benefits attributed to high polyphenol contents biosynthesized by the plant under stress. Studies about correlation of abiotic conditions with anthocyanin profiles and physicochemical features of calafate beneath wild origin environment are not described yet. Hence, this research aimed to evaluate the physicochemical changes, antioxidant activity and anthocyanin content of calafate fruit in relationship to UV solar radiation (W.m^−2^) and air temperature (°C) environment condition during three consecutive years (2017, 2018, 2019). Variations in fruit anthocyanins were determined by comparison between high performance liquid chromatography (HPLC-DAD-ESI)/MSn and CIEL*a*b* colors parameters. Correlations were analyzed by principal component analysis (PCA). Radiation was negatively correlated with fruit size and weight. Physicochemical aspects such as pH, soluble solids, color, total anthocyanins, flavanols and other phenolic compounds were positively correlated with temperature changes. The quantities of monomeric anthocyanins were dependent on both low temperature and global radiation (reaching 20.01 mg g^−1^ FW in calafate fruit). These results constitute a valuable resource to understand the structural and physiological plasticity of calafate in facing climate changes for future domestication research as well as for agri-food industrial application.

## 1. Introduction

Anthocyanins are natural compounds derived from secondary metabolism in plants and they are the second most common bioactive compound present in berry fruits, such as blueberries, raspberries and strawberries [1]. Calafate (*Berberis microphylla* G. Forst) is one of the native perennial bushes widely expanded in Southern climate locations of Chile. Similar to other berries, calafate fruit contains vitamins, minerals, fiber and a particular flavor (acid and sort of astringency) [2]. The importance of this fruit is due to the high polyphenolic compounds, probably related to the extreme environmental conditions where calafate grows and its biological properties [3,4]. The nutritional composition of calafate is described as 4.8 g 100 g^−1^ fatty acids, 8.37 g 100 g^−1^ fiber, 13 g 100 g^−1^ proteins and 70 g 100 g^−1^ of humidity [5], like high delphinidin, delphinidin-derivatives contents and some other anthocyanins [6]. Regarding these compounds, calafate is considered a “superfruit” [2] and has an agro-industrial potential, especially in functional food matter.

In recent studies, the changes in shape, size and structure of leaves of *B. microphylla* have been shown with higher temperatures for genetic improvement purposes [7]. Similarly, the effect of fertilization and irradiance was measured in terms of changes in leaves pigments [8] as well as the ethylene response in calafate fruits after harvest [9]. As described, previous work has only addressed the physical variations in calafate but the physicochemical in relation with climate changes still remain to be studied. Since the chemical structure of anthocyanins in fruit can easily change by variations in particular environmental conditions, it is crucial to understand how these environmental changes influence the anthocyanins pattern in calafate, as well as its correlation with these environmental conditions. In some berries such as *Vaccinum corymbosum*, researchers indicated that light conditions and temperature had an important role on the fruit phenolic compounds [10]. The comparison between different southern Chilean locations has confirmed differences in calafate’s anthocyanins content [2]. Nevertheless, there still have been several constraints around the correlation between years of production and the influence of abiotic stress in anthocyanin contents tackling the climate changes.

Other crucial aspects regarding the fruit quality are the color and aroma. Thus, along with the phenological changes of berries, the phenolic compounds are responsible for conferring the particular characteristics of each berry in terms of color and aroma [11]. It is well known that color derived from anthocyanins in berry fruits appear during the second phase of berry growth (veraison) and continue until the harvest of fruits. However, the mentioned characteristics can also be affected by climate changes, diminishing the fruit quality. Under natural environments the composition of bioactives can also fluctuate. Those changes reflected in anthocyanin concentration are associated with fruit metabolism or other physiological phenomena at the whole plant level and have been studied in other species [12].

*B. microphylla* grows under wild conditions in Chile from the Maule region (34°35′50″ S) to Tierra de Fuego (54°36′48.55″ S) but mainly in the Patagonia zone. This shrub can spring up in a wide range of altitudes, from 0 to 2000 m above the sea [13]. In the Aysén region, *B. microphylla* and other *Berberidaceae* are found from the coast to the Patagonian steppe, with calafate being a characteristic shrub of anthropic grasslands [14]. The climate in Chilean Patagonia is Andean boreal with 1000–1200 mm rainfall per year, mean air temperature of 4.4 °C and global radiation 5000–6000 W.m^−2^ [15]. Under these conditions, calafate blooms from November to December, while veraison to ripening stages occurs between January to March. However, other authors mentioned that calafate phenological stages depend directly on the geographical distribution and altitude. Hence, blooming is earlier in the north of the Aysén region (44°27′27.83″ S, 73°8′14.78″ O) and later in the south (48°28′07″ S, 72°33′33″ O) while, regarding the elevation, the best productive behavior of calafate is in the coast where rainfall is higher [16].

The Intergovernmental Panel on Climate Change (IPCC) predicts 1–3 °C of temperature increasing rainfall and temperatures patterns as a consequence of a decrease in snowfall [17], and changes on UV radiation by alterations of the amounts of ozone, aerosols, and clouds in the atmosphere [18]. This data suggest that climate change could deteriorate crops in general including berry fields by affecting its production and this effect of high temperatures on anthocyanin content is relevant for these berries considering its potential for the food industry. This information has not yet been elucidated and might contribute to planning better management in the domestication to maintain the anthocyanin berries content. Consequently, we hypothesize, as a consequence of extreme UV solar radiation exposure and lower temperatures, the synthesis of phenolic compounds in calafate berries can be highly stimulated. With all this background, the present research aimed to evaluate physicochemical characterization and antioxidant activity and phenolic content of calafate berries as related with the environmental conditions of UV radiation and air temperature from three continuous years (2017, 2018, 2019). We include phenolic identification complimented by analytical methods high performance liquid chromatography (HPLC-DAD-ESI/MS^n^) and CIEL*a*b* color parameters determination. The correlation of color, phytochemicals and antioxidant activity were done by principal component analysis (PCA) method.

## 2. Materials and Methods

### 2.1. Plant Material

During three continuous years (2017, 2018, 2019), ripened calafate berries were obtained between the last week of February and the first week of March, taking into account the recommended harvest time [19]. Fruits (1 kg per year) were collected by hand and detached from 10 random shrubs of a natural population of calafate shrubs near Balmaceda, Coyhaique, Aysén region (45°33′18″ S, 71°50′38.399″ O), 800 m above sea level. Calafate berries were kept on freeze until they were used for analysis.

### 2.2. Chemical Reagents

The analytical-grade reagents (formic acid, acetonitrile, methanol and water), as well as sodium carbonate (Na_2_CO_3_), potassium chloride, the radical 2,2-diphenyl-1-picrylhydracil (DPPH^●^), Trolox, FeCl_3_-6H_2_O, HCl and TPTZ were obtained from Merck (Darmstadt, Germany). Commercial standards of delphinidin 3-glucoside (98.84%) and cyanidin 3-glucoside (99.21%) were obtained from Sigma Aldrich (St. Louis, MO, USA).

### 2.3. Meteorological Data Information

Environmental conditions (UV global radiation and temperature) were studied from Teniente Vidal Station, Coyhaique in Aysén region (45°33′18″ S, 71°50′38.399″ O), where calafate is found in abundance. The information corresponded to three continuous periods of time: 2017 (from October 2016 to March 2017); 2018 (October 2017 to March 2018); and 2019 (from October 2018 to March 2019). The minimum temperature was taken between 8 pm (the day before) and 8 am (the day registered). Similarly, degree days threshold 10 °C and global/accumulated radiation were registered daily. The radiation data corresponds to the monitoring of global and accumulated radiation recorded in the weather station mentioned above, according to their frequency during the day in the mentioned periods.

### 2.4. Sample Preparation

Frozen fruit pulp (100 g calafate berries) was ground, and 50 mg of the resulting powder was poured into 15 mL of 25:24:1 (methanol: water: formic acid) for each sample. The slurry was stirred for 5 min and centrifuged at 10,000× rev.min^−1^ for 10 min. The supernatant was separated in 15 mL tubes. Next, the tubes were merged in ultrasound (1 h) and kept at 4 °C overnight [20]. Independent extracts were prepared for each year’s samples and the aliquots were stored at −20 °C.

### 2.5. Physical and Chemical Parameters

The morphological and chemical estimations were carried out by the use of a bulk of randomized calafate fruits per year. Equatorial and polar diameters of 20 calafate berries and weight in 100 fruits of each year were measured in triplicates. The pH, soluble solids and acidity were obtained from the grounded fruits as indicated in the section of sample preparation. The measurement of the pH was done with a digital pH meter INOLAB series PH7110 (Merck, Darmstadt, Germany) ±0.01 of accuracy. The soluble solids were determined using a SPER SCIENTIFIC series 300,010 refractometer (0.2% Resolution, ±0.2% Accuracy) (Scottsdale, AZ, USA), expressing the results in °Brix. Acidity was determined by titration with NaOH 0.1 M from 0.5 g of crushed sample diluted in 150 mL of distilled water [21]. The color parameters CIEL*a* b*, as well as C* (Color saturation) and °H (Hue angle, tan^−1^ (b/a) were measured as previously described [22], with a PCE-CSM 2 colorimeter (Hunter Lab^®^, Murnau am Staffelsee, Germany). The colorimeter was calibrated with a white disk plate before reading and nine measurements per year were made (*n* = 27).

### 2.6. Identification and Quantification of Anthocyanins, Flavonols and Other Phenolic Compounds

The calafate anthocyanins, flavanols and other phenolic compounds were identified according to their UV spectra, order of elution, and MS/MS fragmentations, as previously described for similar acquisition conditions. The HPLC-DAD-ESI/MSn analyses were carried out on an Agilent HPLC 1100 series equipped with a photodiode array detector and a mass detector in series (Agilent Technologies, Waldbronn, Germany). The equipment consisted of a binary pump (model G1312A), an autosampler (model G1313A), a degasser (model G1322A) and a photodiode array detector (model G1315B). The HPLC system was controlled by ChemStation software (Agilent, version 08.03). The mass detector was an ion trap spectrometer (modelG2445A) equipped with an ESI interface and controlled by LCMSD software (Agilent, version 4.1). The ionization conditions were adjusted to 350 °C and 4 kV for capillary temperature and voltage, respectively. The nebulizer pressure and flow rate of nitrogen were 65.0 psi and 11 L min^−1^, respectively. The full-scan mass covered the range from m/z 100 up to m/z 1200. Collision-induced fragmentation experiments were performed in the ion trap using helium as the collision gas, with voltage ramping cycles from 0.3 up to 2 V. MS data were acquired in the positive ionization mode for anthocyanins. MS^n^ was carried out in the automatic mode on the more abundant fragment ion in MS^(n−1)^ [23].

The quantification of anthocyanins, flavanols and other phenolic compounds were held with the aliquots aforementioned in the sample preparation section, filtered through a 0.22 μm PVDF membrane (Millex V13, Millipore, Bedford, MA, USA). The quantitative analysis was carried out with similar conditions as reported before [24], using an Agilent Technologies 1220 Infinity Liquid Chromatograph equipped with an autoinjector (G1313, Agilent Technologies, Santa Clara, CA, USA) and a Diode Array Detector (1260, Agilent Technologies, Santa Clara, CA, USA). The chromatographic system Phenomenex (Torrens, CA, USA) was adapted with a Luna 5 μm C18, 100 Å column (250 × 4.6 mm), and Security Guard Cartridges PFD C18 4 × 3.0 mm) were used. The chromatographic separation was performed with 1% formic acid and methanol as mobile phases A and B, respectively, with a flow rate of 0.9 mL min^−1^. The linear gradient (time, %B) was as follows: (0%, 15%); (20%, 30%); (30%, 40%); (35%, 60%); (40%, 90%); (44%, 90%), and back to initial conditions for column stabilization (10 min) until 70 min. The analyses procedure was based on calibration curves using the following standards: cyanidin 3-*O*-glucoside at 520 nm for anthocyanins, quercetin 3-*O*-rutinoside at 360 nm for flavanols, 3-O-caffeoylquinic acid at 320 nm for cinnamic and derivatives and expressed in mg g^−1^. The calibration curve for cyanidin standard was 0.01, 0.1, 0.5, 1, 2 ppm (linear regression y = 12618 ×+ 1316.3, limit of detection 0.10 and limit of quantification 0.31, R^2^ 0.99) concentration of standards for 2mM concentration of the samples (1:10). The parameters were considered in the equipment (confidence level 99%) and threshold as default.

### 2.7. Antioxidant Capacity Determination

The antioxidant activity by free radical scavenging was performed by DPPH^●^ (2,2-diphenyl-1-picrylhydrazyl) while the antioxidant reducing capacity was carried out by FRAP (Ferric Reducing Antioxidant Power Assay). DPPH^●^ assay was carried out with 100 µL of the extract and 2.9 mL of the DPPH^●^ solution, constantly shaken and then incubated in darkness for 1 h. The reading was taken at 515 nm and results expressed in µmol Trolox equivalents g^−1^ of fresh weight sample [25]. In the FRAP assay we used 30 µL of the extract (per sample), 300 µL of distilled water and the addition of 3000 µL of FRAP reagent (FeCl_3_-6 H_2_O 20 mM, TPTZ 10 mM and acetate buffer 0.3 mM) incubated at 37 °C for 30 min [26]. All estimations were done at 595 nm absorbance in the spectrophotometer described above and expressed in μmol Trolox equivalents g^−1^ of fresh weight sample.

### 2.8. Statistical Analysis

Data were subjected to analysis of variance (ANOVA) at *p* < 0.05 level of significance. Mean comparison was carried out by Tukey’s test with a significance level of 0.05. PCA was employed by mean centered data based on the eigen values to determine the correlation between variables and the discrimination of anthocyanin contents of calafate and phytochemical characteristics in different conditions of global radiation and air temperature using R software [27] by FactoMineR and ggplot2 [28] package.

## 3. Results and Discussion

In the results ahead we intend to provide information regarding the relationship of compounds present in calafate berries and the changes they may suffer due to the effect of climate change (solar radiation and temperature). Environmental constraints due to climate change can positively affect the production of bioactive compounds, in some cases leading to detriment in quality parameters such as fruit weight. Plants produce specific metabolites as protective mechanisms during the acclimatization. These two processes improve the plant plasticity to cope with the stress conditions and reduce the impact in the development as well as productivity. Knowing the interaction between environmental factors and physical-chemical parameters is a key approach to elucidate the reaction of calafate berries to climate change and can be a useful tool for domestication, support of agronomic management programs and genetic improvement. Three years analyzing calafate berries harvested in a native origin allows us to confirm the plasticity of this berry to adapt under particular solar radiation and temperature conditions at the southern zone of Chile.

### 3.1. Variation in Environmental Conditions

Environmental factors stimulate the production of secondary metabolites in berries. To address this issue in calafate, we considered temperature and UV solar radiation since their relation to anthocyanins production is well known. The data set of environmental parameters were obtained from the closest agrometeorological station, as some authors recommend [29]. The values considered for temperature were the minimum and maximum/day and degree days. Regarding the solar UV radiation indexes, the maximum radiation per day since October to March from three continuous periods of time was examined (2016–2017, 2017–2018 and 2018–2019, called Year 2017, 2018 and 2019, respectively). The months mentioned concur with the crucial stages of calafate berry production bud-break, veraison and harvest in the location studied. This work was based on what was previously described for calafate in the area of Aysén [16], since in terms of phenology these periods are crucial for bioactive compounds production. Despite the fact that in other regions such as Araucanía the phenological stages of calafate are brought forward to July–December [30], in the southernmost part of the country (Magellan region) it is delayed to October–March [31]. This variation is due to the geographical area where the fruits were harvested.

**Figure 1:**
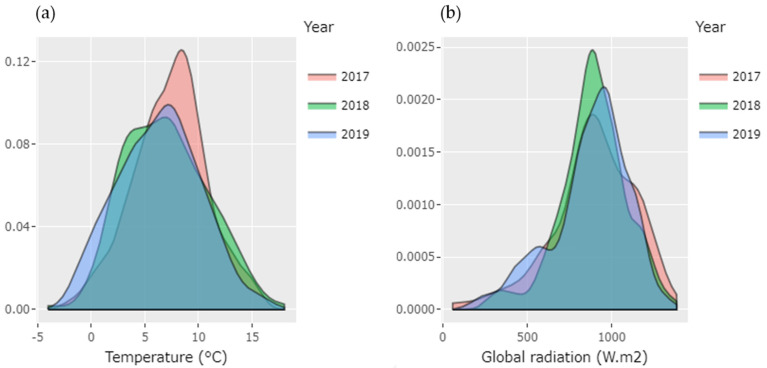
Minimum Temperature in °C (**a**) and maximum UV solar radiation/day (W m^−2^) (**b**) during three consecutive years (2017–2019) of the experimental period with calafate berries in Coyhaique.

Considering the temperature, the coldest time was during 2019 (Figure 1a) and the warmest was 2018. The range of minimum temperature was −5 to 16 °C and the mean peak was between 7 to 10 °C, similar to registers for the last decade [15] (whole register for maximum and minimum temperature and degree days are in Supplementary Materials S1 and S2, correspondingly). The degree days are an important parameter; it is involved in the plant growth, development and production and is also considered relevant for the assessment of the impact of the climate change [32]. This parameter was estimated using a threshold of 10 °C [33]. Although the year 2017 was neither the warmest nor the hottest, the degree days (DD) parameter was higher in this year (454.5 DD). Surprisingly, the degree days decreased within the period studied. Thus, 2018 recorded 442 DD. In year 2019 the accumulated degree days were 412.6 °C even though the year 2019 reported cold temperatures in the day-to-day analysis reported above. In crops such as grapes, it is described that cold periods during the veraison help the accumulation of secondary metabolites [11]. Although this work studied how low temperatures influenced the production of anthocyanins, it is important to note that high temperatures could significantly increase the problems generated by thermal and radiation stress in the production of berries such as calafate.

For all years studied, the global solar radiation was notably intense in December; the highest value was 1082 W m^−2^ (during 2017), while minimum values were observed in March during all the study time (Supplementary S1). Authors mentioned the optimum radiation recommended for berries such as grape during bud break until harvest is 700 W m^−2^ to have a good production with a considerable quantity of polyphenols [34]. By density distribution shown (Figure 1b), we confirmed that 2017 had the highest frequencies of UV radiation per day. In addition, the cumulative maximum radiation increased by year from 5424.1 to 6001.1 W m^−2^. Even if the differences by year are not significant in statistical terms, this tendency of increasing corresponds to the information brought by IPCC, estimating that UV radiation is rising gradually due to climate change [17]. This increase in radiation represents almost 4% accumulated in the three years of study. The analysis of temperature and radiation data show the variation from previous years reported [35]. It is described that the climate change would register an increase of the global temperature itself that has been more notorious in the zones closer to the poles [36]. Regarding radiation, the weakening of the ozone layer could determine a high radiation in the southern areas of Chile [17], and as a consequence this condition could affect the polyphenols production in calafate.

### 3.2. Physicochemical Parameters

The morphological parameters bring a general view of the changes in calafate fruits of three consecutive years in order to characterize them. Our results are described in Table 1. Calafate 2018 presents significantly lower values of weight, polar and equatorial diameter compared to the first and third year. As we noticed, this is a result of thermal amplitude observed that year, since low temperatures for long periods cause small berries to be produced by plants [37]. Ranges of pH 3.6–3.7 are in agreement with the last year’s publication [21] while in acidity and soluble solids concerning 2% and 27.2 °Brix, data showed the highest values in the first year of this study. That year stands out because it presents lower temperature and higher radiation favoring stress in plants. It seems that the plant responds to the synthesis of sugars and the decrease of organic acids more because of these factors, since 2019 has a larger berry size, but the acidity is also lower than it was in 2018.

### 3.3. Analysis of CIEL*a*b* Values

Color can be affected by changes in pH, seasonal changes and environmental factors, and it is also known that it is an indicator of quality food as well as providing an estimation of the amount of antioxidant compounds contained in fruits, particularly in berries [38]. To determine the influence of color on calafate anthocyanins, the CIEL*a*b* space was used to compare color perception, hue angle, lightness and saturation. We found significant differences between the calafate fruits corresponding to 2017, 2018 and 2019 in all parameters, however, b* describes a direct relationship with pH and the same relation is indirect with a*. Color is an interesting parameter to correlate to anthocyanin content, however, it is not very easy to give an interpretation to the CIEL*a*b* colorimetric analyses, mainly because data about calafate berries are not available in literature. In addition, results in this analysis are influenced by pH, temperature, oxygen content and also the observed hue angle. Colorimetric parameters of calafate berries of three years are reported in Table 2. The L* value (29.47–33.78) represents the darkness of calafate samples. The a* value, in the red region of the colorimetric space, ranged from 0.65–1.98, all of them positives, and the b* value negatives (−0.08 to −2.21), in the yellow region of the colorimetric space, indicated the red-dark appearance of calafate relating with the high anthocyanin content. The values obtained in this study for calafate are comparable with blueberry [22] results considering that both berries are dark in their aspects; nonetheless, the b* parameter confirms that calafate is even darker. The colorimetric parameters of a* and b* were probably the result of the influence of thermal stress by the thermal amplitude, but not by the low temperatures according to our hypothesis. It is evident that the dark purple color of calafate berries comes from anthocyanins, and we confirmed it in this study.

### 3.4. Comparison of Calafate Anthocyanin Profile and Other Phenolic Compounds

The separation and quantification of individual monomeric anthocyanins by HPLC-DAD brought us the main group of characteristic calafate anthocyanins at 520 nm. The range was 14–20 mg g^−1^ (Table 3). These results are comparable with those obtained for calafate fruits (15 mg g^−1^) in Argentinean Patagonia [39]. The location and environmental conditions are important because the quantification in other references for the same Chilean berry related to the anthocyanin content was lower (9.4 mg g^−1^) [20]. Eleven anthocyanidins were detected (Chromatogram is showed in Supplementary S3). We found similar calafate anthocyanins as detected and reported before [40]. In this research, we quantified seven monomeric anthocyanins as being predominant: Delphinidin 3-glucoside, Petunidin 3-rutinoside, Petunidin 3-glucoside and Malvidin 3-glucoside, while the minority were: Cyanidin 3-glucoside, Malvidin 3-rutinoside and Peonidin 3-glucoside. No previous data regarding the relationship of particular anthocyanins of calafate with UV radiation and temperature in natural origin have been published before. As Fernandes de Oliveira reported, the three glycoside conjugate anthocyanins are the majority due to the temperature and pH of the calafate berry extract as it happens on the grapevine [11]. Furthermore, in this research the differences between the quantities of the aforementioned calafate compounds can be attributed to the studied environmental factor, considering the correlation of temperature and anthocyanins described before. The 2018 sample had the highest frequency of hot days and this period of time presented the higher quantification of anthocyanin. All the monomeric anthocyanins showed the same behavior except for peonidin 3-glucoside, which decreased in this period of time.

**Figure 2:**
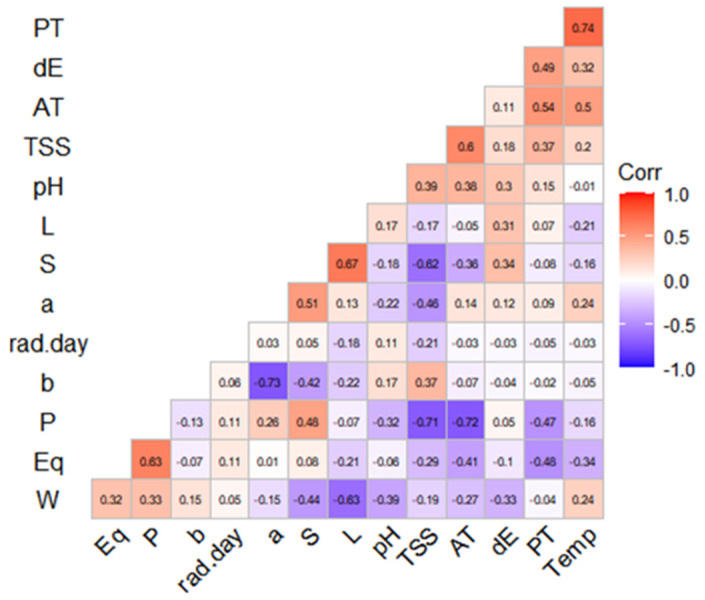
Matrix of correlation between UV solar radiation and temperature with physicochemical parameters of calafate berries. PT, polyphenols, dE, color differential (ΔE); AT, sum of individual anthocyanins; TSS, total soluble solids; pH, potential of Hydrogen; L, luminosity (CIEL*a*b*); S, period of time (2017, 2018, 2019); a and b, chroma in CIEL*a*b* components, P, polar diameter; Eq, equatorial diameter; W, weight; Temp, temperature; rad.day, maximum radiation per day.

The higher the anthocyanin content was the lower the b* value was, denoting the negative correlation between these two parameters (Figure 2) and relating the a* from green to red. Although this is somewhat surprising since the intense red of the calafate should have a high b* component as well, as it becomes a red-purple-blue. Calafate, even when tackling climate change, is still a pigment producer by excellence. The use of thermal or solar radiation stress could even be considered to stimulate anthocyanin production in calafate to cover the demand of the industry. In terms of flavanols, as described before [6], myricetin, quercetin, isorhamnetin derivatives were detected. However, in this research we considered the sum of all flavanols and other phenolic compounds detected by HPLC-DAD and contained in calafate samples per year. Thus, phenolic compound quantities (other than anthocyanins) (2.69–3.59 mg g^−1^ sample FW) were similar to the results reported by López and colleagues [20]. The behavior of flavanols can be attributed to UV radiation combined with the water deficit. Even if we did not consider that last parameter, some studies have confirmed it [41]. Comparing the data of the sum of anthocyanins versus the sum of flavanols and other phenolic compounds, we found that as the anthocyanins were high, the flavanols and other phenolic compounds were low (20.0 mg g^−1^ and 2.69 mg g^−1^, respectively, taking into account the year of 2018). This apparent lack of correlation between these compounds can be attributed to a combination of radiation and temperature that promotes the production of anthocyanins, but high temperatures could diminish the flavanol quantities [41].

#### Antioxidant Capacity in Calafate Berries

Calafate, like other native berries such as maqui, are good sources of polyphenols (anthocyanins, flavanols and other phenolic compounds included) [4] and those compounds have been widely tested for their high antioxidant capacity and benefits to human health. Thus, the calafate berry extracts revealed the best antioxidant reaction for 2017 and is shown in Table 4 (4.07 and 41.55 μmol Trolox g^−1^ FW for FRAP and DPPH, respectively). The year of 2018 had the lowest antioxidant capacity regarding to FRAP method but differed for DPPH due to the difference of way of action in each method (reducing and scavenging radicals in order). This implies that there is a direct relationship between the sum of anthocyanins and flavanols described in the previous section and the antioxidant capacity, observing how it was influenced by the high temperature and high radiation in 2018 and therefore presented a higher antioxidant capacity. As an experimental for calafate and Chilean strawberry, Lopez and colleagues reported slightly higher values for DPPH assay (51 μmol Trolox g^−1^ DW), although it was performed on dry matter.

### 3.5. Correlations

**Figure 3:**
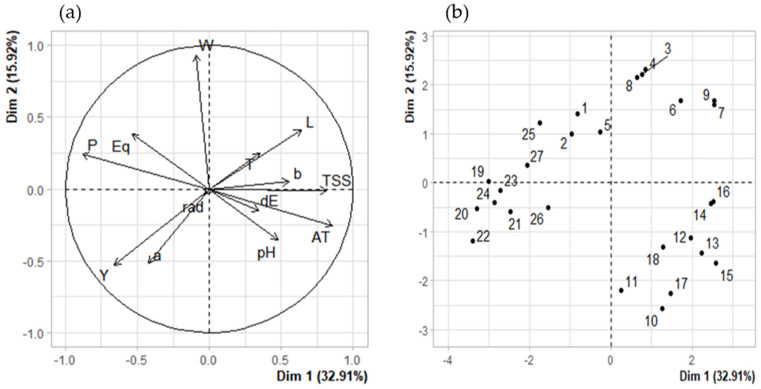
Principal component analysis (PCA) of physical and chemical variables of calafate berry extracts. P, polar diameter; Eq, equatorial diameter; W, weight; L, *a, *b CIEL*a*b* components; dE, ΔE; TSS, total soluble solids; AT, Sum of individual anthocyanins; T, temperature; rad, maximum radiation per day. (**a**) PCA of variables. (**b**) PCA of individuals.

To study the reasons of physical and chemical changes in calafate fruits, we correlated the environmental parameters of UV radiation and temperature. A correlation matrix (Figure 2) to analyze the two climate factors and physic-chemical calafate parameters by Spearman’s test was used. The PCA analysis was performed for 13 main traits (weight, equatorial and polar diameter, pH, *L, *a, *b, ΔE, total soluble solids, sum of anthocyanins, radiation, temperature and Year). PC1 retained 32.9% of the data variation while PC2 retained 15.9%, representing all traits as vectors in the biplot below (Figure 3a), and the length of vectors show how well represented the variables are in this plot. Anthocyanin contents (AT) showed moderate correlation with Temperature (T), similar to the reported results a while ago for grapes [29]. By the analysis, we can also determine that anthocyanins and other phenolic compounds in calafate are not directly related with year of collection (Y). It explains that even if we consider three different years’ samples, the bioactive contents vary as the environmental parameters change yearly. The color parameters were correlated with temperature and negatively correlated less significantly with UV radiation (Figure 3a). The analysis brought the higher correlation (0.74) between phenolic compounds and temperature, while negative correlations were established between soluble solids and polar diameter of calafate fruits (−0.72). It has been described that abiotic factors affect morphological aspects of berry production [34], as well as their nutritional and bioactive compound contents [29]. This research confirms the results reported by Del Castillo regarding phenolic compounds. Nonetheless, in our research the morphological aspects of calafate show a strong relationship with temperature but less with UV global radiation. On the other hand, we also found that quantities of anthocyanins were strongly disfavored when temperature stress was considered as the unique factor. Consequently, the combination of two abiotic factors (UV global radiation and temperature) less often affected physical parameters such as fruit size (Eq, P) and weight (W) (Figure 3). Regarding pH and total soluble solids (TSS), these parameters were correlated with color parameters (*L,*a,*b) with the sum of individual anthocyanins (AT) and they also showed positive correlation with Temperature. Nonetheless, they were also dependent of the fruit maturation, as mentioned before by other researchers [8].

Solar radiation exposure had no influence on parameters such as size (P, Eq) and weight (W) (Figure 2), but it was slightly negative correlated with color parameters (*L, *b, dE). That influence was confirmed by the PCA analysis (Figure 3a). In response to environmental limitations such as low temperatures, the calafate displayed structural and physiological plasticity that allowed it to adapt to different temperatures caused by the diurnal and seasonal rhythms associated with anthocyanin contents growing wild, although facing UV radiation was not a determinant factor in this research. This could be caused by the previous adaptation of the calafate to the extreme condition of the constant high radiation exposure. Anthocyanins, including delphinidin and cyanidins, were specifically downregulated by cold or by UV radiation [11]. Interestingly, these compounds are similar to ones reported in this research (Table 3). Non-colored polyphenols were not considered in this study. The correlation circle explaining the PCA of variables (Figure 3a) showed that it is mandatory to explain that in the PCA of individuals, the numbers from 1–9 correspond to 2017, 10–18 data from year 2018, and finally 19–27 with 2019, underlining that even with the differences considered regarding climate factors and morpho-chemical parameters as they are grouped, this happens with saffron samples grouped by location [38].

## 4. Conclusions

In general, the global solar radiation and temperature from three consecutive years of study were not determinant for the physicochemical features in calafate but caused an effect in the anthocyanin profile of calafate. Being that calafate was considered as a potential for agronomic domestication and as a health-promoting food, this work contributes to a better understanding of how anthocyanins profiles, antioxidant capacity and other physicochemicals facing UV global radiation and temperature factors. Furthermore, these results contribute to understand the adaptation of calafate berries under changing climate conditions, focusing on the food security (due to its condition of safe, nutritious and healthy berries to meet the nutritional needs) and as a source of bioactive compounds. The next step is to start the domestication of calafate, taking into consideration what is presented here and also to study the changes at a physiological and molecular level as an effect of climate change (stressors such as UV radiation and temperature) that occur in the calafate berries.

## Figures and Tables

**Table 1:** Physicochemical parameters of Calafate berries of three consecutive years (2017, 2018 and 2019).

Sample	Weight (g)	Equatorial Diameter (mm)	Polar Diameter (mm)	pH	Soluble Solids (°Brix)	Acidity (%)
Calafate 2017	0.60 ± 0.02 ^c^	9.9 ± 0.04 ^ab^	9.0 ± 0.06 ^a^	3.61 ± 0.03 ^ab^	27.2 ± 0.10 ^b^	2.0 ± 0.07 ^a^
Calafate 2018	0.50 ± 0.01 ^a^	8.5 ± 0.38 ^a^	7.8 ± 0.14 ^a^	3.66 ± 0.01 ^a^	26.8 ± 0.02 ^b^	1.0 ± 0.03 ^b^
Calafate 2019	0.54 ± 0.06 ^b^	10.3 ± 0.16 ^b^	8.4 ± 0.50 ^c^	3.7 ± 0.01 ^b^	22.7 ± 0.37 ^a^	0.9 ± 0.03 ^b^

Different letters in the same column mean significant differences at (*p* ≤ 0.05).

**Table 2:** CIEL*a*b* color parameters of calafate berries evaluated during three consecutive years: 2017, 2018 and 2019.

Year/Color Parameter	L*	a*	b*	c*	H°	ΔE
2017	29.47 ± 7.5 ^a^	0.61 ± 0.02 ^a^	−0.08 ± 0.21 ^a^	1.59 ± 0.14 ^a^	7.71 ± 0.11 ^a^	2.35 ± 0.05 ^a^
2018	35.12 ± 6.20 ^b^	1.05 ± 0.18 ^a^	−0.88 ± 0.07 ^ab^	2.48 ± 0.43 ^ab^	39.79 ± 0.71 ^b^	4.41 ± 0.59 ^b^
2019	33.78 ± 5.43 ^b^	1.98 ± 0.08 ^b^	−2.21 ± 0.16 ^b^	3.36 ± 0.08 ^b^	47.98 ± 0.49 ^c^	3.74 ± 0.54 ^a,b^

Data are expressed as mean. Different subscript letters in the same column mean significant differences at (*p* ≤ 0.05).

**Table 3:** Main anthocyanins, flavanols and other phenolic compounds detected by high performance liquid chromatography (HPLC) and found in the Calafate berry over three consecutive years.

Compounds	2017	2018	2019
Delphinidin 3-glucoside	10.68 ^b^	11.60 ^a^	9.78 ^a,b^
Cyanidin 3-glucoside	0.20 ^a^	0.42 ^b^	0.25 ^a,b^
Petunidin 3-glucoside	3.08 ^b^	3.38 ^b^	1.73 ^a^
Petunidin 3-rutinoside	4.17 ^b^	4.44 ^b^	2.47 ^a^
Peonidin 3-glucoside	0.03 ^a,b^	0.02 ^b^	0.33 ^a^
Malvidin 3-glucoside	0.35 ^c^	0.03 ^a^	0.14 ^b^
Malvidin 3-rutinoside	0.10 ^b^	0.12 ^c^	0.07 ^a^
Sum of anthocyanins	18.62 ^b^	20.0 ^a^	14.77 ^c^
Sum of flavanols and other phenolic compounds	3.42 ^b^	2.69 ^c^	3.59 ^a^
Sum of phenolic compounds	22.04 ^b^	22.69 ^a^	18.36 ^c^

The values are expressed in mg g^−1^. Different superscript letters in the same row mean significant differences at (*p* ≤ 0.05).

**Table 4:** Antioxidant capacity by Ferric Reducing Antioxidant Power Assay (FRAP) and 2,2-diphenyl-1-picrylhydrazyl (DPPH^●^) assays of calafate berry for three consecutive years (2017, 2018 and 2019).

Year	FRAP	DPPH^●^
2017	4.07 ± 0.03 ^a^	41.55 ± 0.96 ^b^
2018	3.53 ± 0.07 ^a^	36.47 ± 0.94 ^a^
2019	4.12 ± 0.02 ^a^	35.44 ± 0.11 ^a^

FRAP (µmol Trolox g^−1^ of sample FW), DPPH^●^ (µmol Trolox g^−^^1^ of sample FW). Different letters in the same column mean significant differences at (*p* ≤ 0.05).

## Supplementary Materials

The following are available online at https://www.mdpi.com/2304-8158/10/1/196/s1, S1: Temperature (maximun and minimum) registered day-to-day in Coyhaique, Aysen region during three consecutive periods of time A. 2016–2017 B. 2017–2018 and C. 2018–2019. S2: Degree days base 10 registered in Coyhaique, Aysen región during three consecutive periods of time Figure S3: Calafate anthocyanins identified by chromatography. (A) HPLC chromatogram detected at 520 nm for the individual anthocyanin quantification. (B) HPLC chromatogram detected at 360 nm (C) Identification of main phenolic compounds by HPLC-DAD-ESI/MS^n^.

## References

[1] Khoo H.E., Azlan A., Tang S.T., Lim S.M. (2017). Anthocyanidins and anthocyanins: Colored pigments as food, pharmaceutical ingredients, and the potential health benefits. Food Nutr. Res..

[2] Mariangel E., Reyes-Diaz M., Lobos W., Bensch E., Schalchli H., Ibarra P. (2013). The antioxidant properties of calafate (Berberis microphylla) fruits from four different locations in southern Chile. Ciencia Invest. Agrar..

[3] Manosalva L., Mutis A., Urzúa A., Fajardo V., Quiroz A. (2016). Antibacterial Activity of Alkaloid Fractions from Berberis microphylla G. Forst and Study of Synergism with Ampicillin and Cephalothin. Molecules.

[4] Salehi B., Selamoglu Z., Sener B., Kilic M., Jugran A.K., De Tommasi N., Sinisgalli C., Milella L., Rajkovic J., Morais-Braga M.F.B. (2019). Berberis Plants-Drifting from Farm to Food Applications, Phytotherapy, and Phytopharmacology. Foods.

[5] Araya M. (2010). Estudio Preliminar de La Composición Química y El Valor Nutricional de Frutos Regionales de Interés Econó-mico y Sociocultural de Magallanes.

[6] Ruiz A., Hermosín-Gutiérrez I., Mardones C., Vergara C., Herlitz E., Vega M., Dorau C., Winterhalter P., Von Baer D. (2010). Polyphenols and Antioxidant Activity of Calafate (Berberis microphylla) Fruits and Other Native Berries from Southern Chile. J. Agric. Food Chem..

[7] Radice S., Arena M.E. (2015). Environmental effect on the leaf morphology and anatomy of Berberis microphylla G. Forst. Int. J. Plant Biol..

[8] Arena M.E., Pastur G.M., Lencinas M.V., Soler R., Bustamante G. (2020). Changes in the leaf nutrient and pigment contents of Berberis microphylla G. Forst. in relation to irradiance and fertilization. Heliyon.

[9] Rodoni L.M., Feuring V., Zaro M.J., Sozzi G.O., Vicente A.R., Arena M.E. (2014). Ethylene Responses and Quality of Antioxi-dant-Rich Stored Barberry Fruit (Berberis Microphylla). Sci. Hortic..

[10] Yan X., Yan J., Pan S., Yuan F. (2020). Changes of the Aroma Composition and Other Quality Traits of Blueberry ‘Garden Blue’ during the Cold Storage and Subsequent Shelf Life. Foods.

[11] De Oliveira A.F., Mercenaro L., Del Caro A., Pretti L., Nieddu G. (2015). Distinctive Anthocyanin Accumulation Responses to Temperature and Natural UV Radiation of Two Field-Grown Vitis vinifera L. Cultivars. Molecules.

[12] Ferreira S.S., Silva P., Silva A.M., Nunes F.M. (2020). Effect of harvesting year and elderberry cultivar on the chemical composition and potential bioactivity: A three-year study. Food Chem..

[13] Pino M.T., Pérez R., Vergara C., Zamora O., Dominguez E. (2019). Michay: Berry Nativo de Amplia Distribución Con Metabo-litos de Interés Para La Industria de Alimentos. Inf. INIA La Platina.

[14] Silva F. (2013). Flora agropecuaria de Aysén. Servicio de Agricultura y Ganadería.

[15] Hepp K., Stolpe N.B. (2014). Caracterización y Propiedades de Los Suelos de La Patagonia Occidental (Aysén).

[16] Salinas S., Gómez N., Riquelme Espergue F., Acuña Aroca B., Díaz V. (2019). Manual de Productos Forestales No Madereros (PFNM). Proyecto Manejo Sustentable de La Tierra Comuna de Coyhaique.

[17] Magrin G.O., Marengo J.A., Boulanger J.-P., Buckeridge M.S., Castellanos E., Poveda G., Scarano F.R., Barros V., Field C., Dokken D. (2014). Central and south america. Impacts, Adaptation, and Vulnerability.

[18] Bais A.F., McKenzie R.L., Bernhard G., Aucamp P.J., Ilyas M., Madronich S., Tourpali K. (2014). Ozone depletion and climate change: Impacts on UV radiation. Photochem. Photobiol. Sci..

[19] Arribillaga G.P. (2001). Domesticación del Calafate (Berberis buxifolia L.) Para Fines Agroindustriales.

[20] López M.D., Baenas N., Retamal-Salgado J., Zapata N., Moreno D.A. (2018). Underutilized Native Biobío Berries: Opportunities for Foods and Trade. Nat. Prod. Commun..

[21] Romero Román M., Noriega Vásquez F., Farías Villagra M., Belchi L., Jara Zapata P., Vera Flores B. (2019). Nuevas Fuentes de Antioxidantes Naturales: Caracterización de Compuestos Bioactivos En Cinco Frutos Nativos de Chile. Perfiles.

[22] Cesa S., Carradori S., Bellagamba G., Locatelli M., Casadei M.A., Masci A., Paolicelli P. (2017). Evaluation of processing effects on anthocyanin content and colour modifications of blueberry (*Vaccinium* spp.) extracts: Comparison between HPLC-DAD and CIELAB analyses. Food Chem..

[23] Salar F.J., Agulló V., García-Viguera C., Domínguez-Perles R. (2020). Stevia vs. Sucrose: Influence on the Phytochemical Content of a Citrus–Maqui Beverage—A Shelf Life Study. Foods.

[24] Agulló V., Villaño D., García-Viguera C., Domínguez-Perles R. (2020). Anthocyanin Metabolites in Human Urine after the Intake of New Functional Beverages. Molecules.

[25] Bondet V., Brand-Williams W., Berset C. (1997). Kinetics and Mechanisms of Antioxidant Activity using the DPPH.Free Radical Method. LWT.

[26] Benzie I.F.F., Strain J.J. (1996). The ferric reducing ability of plasma (FRAP) as a measure of “antioxidant power”: The FRAP assay. Anal. Biochem..

[27] RStudio Team (2020). RStudio: Integrated Development for R..

[28] Wickham H. (2011). Ggplot2. Wiley Interdiscip. Rev. Comput. Stat..

[29] Del-Castillo-Alonso M.Á., Castagna A., Csepregi K., Hideg É., Jakab G., Jansen M.A., Jug T., Llorens L., Mátai A., Mar-tínez-Lüscher J. (2016). Environmental Factors Correlated with the Metabolite Profile of Vitis Vinifera Cv. Pinot Noir Berry Skins along a European Latitudinal Gradient. J. Agric. Food chem..

[30] Cardenas A. (2012). Identificación e Incidencia de Puccinia Meyeri-Albertii P. Magn. en Calafate (Berberis microphylla G. Forst.) y Michay (Berberis darwinii Hook.) en el Llano Central de la Región de La Araucanía.

[31] Saavedra J., Pino M.T., Zamora O., Ojeda A., Leod C.M., Aguila K. (2017). Análisis de diversidad genética del calafate en Maga-llanes. Inf. INIA Kampenaike.

[32] Hykkerud A.L., Uleberg E., Hansen E., Vervoort M., Mølmann J., Martinussen I. (2018). Seasonal and Yearly Variation of Total Polyphenols, Total Anthocyanins and Ellagic Acid in Different Clones of Cloudberries (*Rubus Chamaemorus* L.). Angew. Bot..

[33] Gentilucci M. (2018). Grapevine Prediction of End of Flowering Date.

[34] Nunes N., Leite A., Castro C. (2016). Phenology, Reproductive Biology and Growing Degree Days of the Grapevine ‘Isabel’(Vitis Labrusca, Vitaceae) Cultivated in Northeastern Brazil. Braz. J. Biol..

[35] Portal de Servicios Climáticos—Dirección Meteorológica de Chile. https://climatologia.meteochile.gob.cl/application/mensuales/climatMensualDatosDiarios/450004/2019/3.

[36] Pellicciotti F., Ragettli S., Carenzo M., McPhee J. (2014). Changes of glaciers in the Andes of Chile and priorities for future work. Sci. Total Environ..

[37] Di Vittori L., Mazzoni L., Battino M., Mezzetti B. (2018). Pre-harvest factors influencing the quality of berries. Sci. Hortic..

[38] Anuar N., Taha R., Mahmad N., Mohajer S., Musa S.A.N.C., Abidin Z.H. (2017). Correlation of colour, antioxidant capacity and phytochemical diversity of imported saffron by principal components analysis. Pigment. Resin Technol..

[39] Chamorro M.F., Reiner G., Theoduloz C., Ladio A.H., Schmeda-Hirschmann G., Gómez-Alonso S., Jiménez-Aspee F. (2019). Polyphenol Composition and (Bio)Activity of Berberis Species and Wild Strawberry from the Argentinean Patagonia. Molecules.

[40] Brito A., Areche C., Sepulveda B., Kennelly E.J., Simirgiotis M.J. (2014). Anthocyanin Characterization, Total Phenolic Quantification and Antioxidant Features of Some Chilean Edible Berry Extracts. Molecules.

[41] Guan L., Dai Z., Wu B.-H., Wu J., Merlin I., Hilbert G., Renaud C., Gomès E., Edwards E., Li S.-H. (2016). Anthocyanin Bio-synthesis is Differentially Regulated by Light in the Skin and Flesh of White-Fleshed and Teinturier Grape Berries. Planta.

