# Supplementary figures and images for: Native Species Facing Climate Changes: Response of Calafate Berries to Low Temperature and UV Radiation
Source: Foods. 2021 Jan 19;10(1):196. doi: 10.3390/foods10010196 (PMC7835903; doi:10.3390/foods10010196)

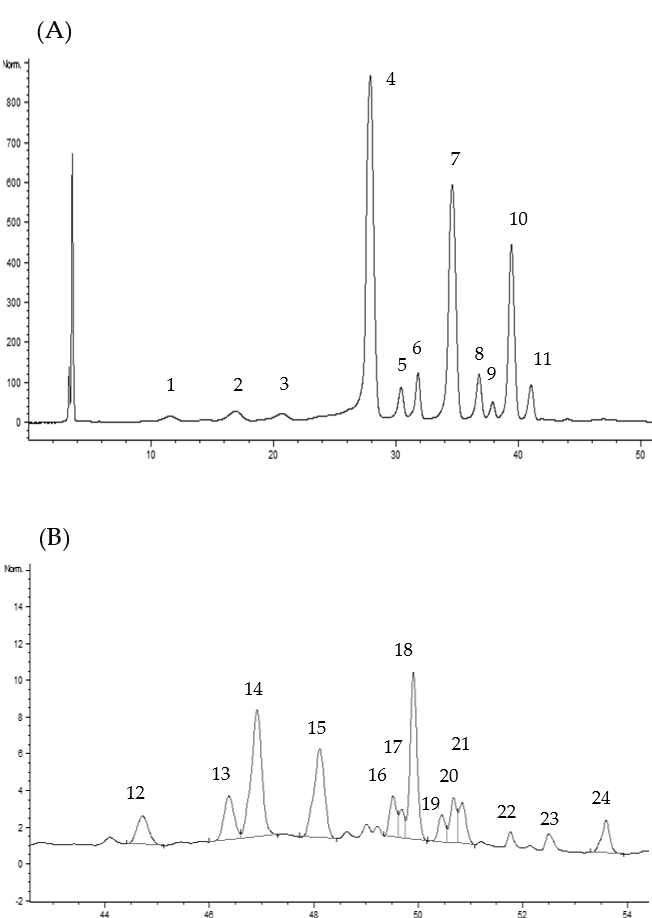

Supplement: Supplementary file 1 [file foods-10-00196-s001.zip › S3_A_B.tif]

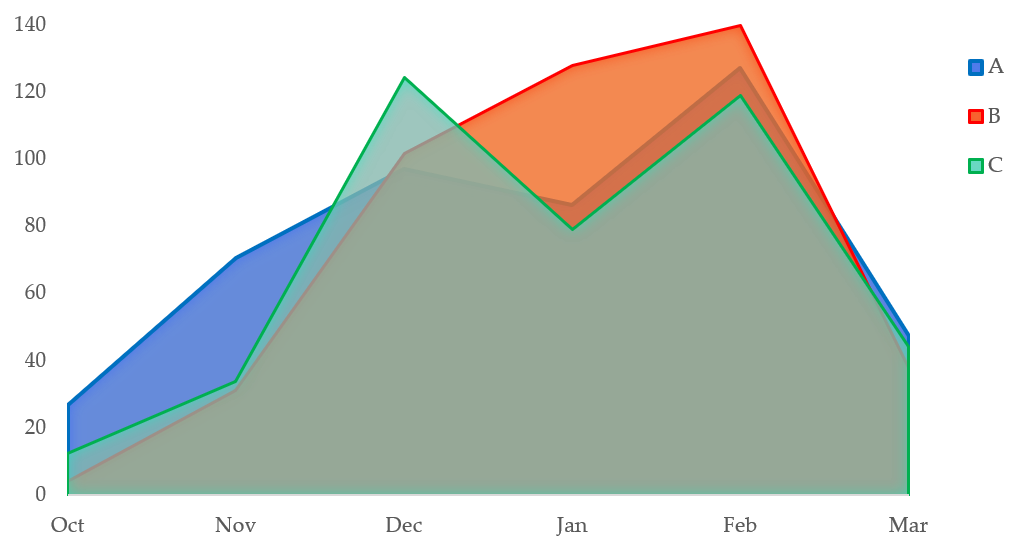

Supplement: Supplementary file 1 [file foods-10-00196-s001.zip › S2. Degree days.tif]

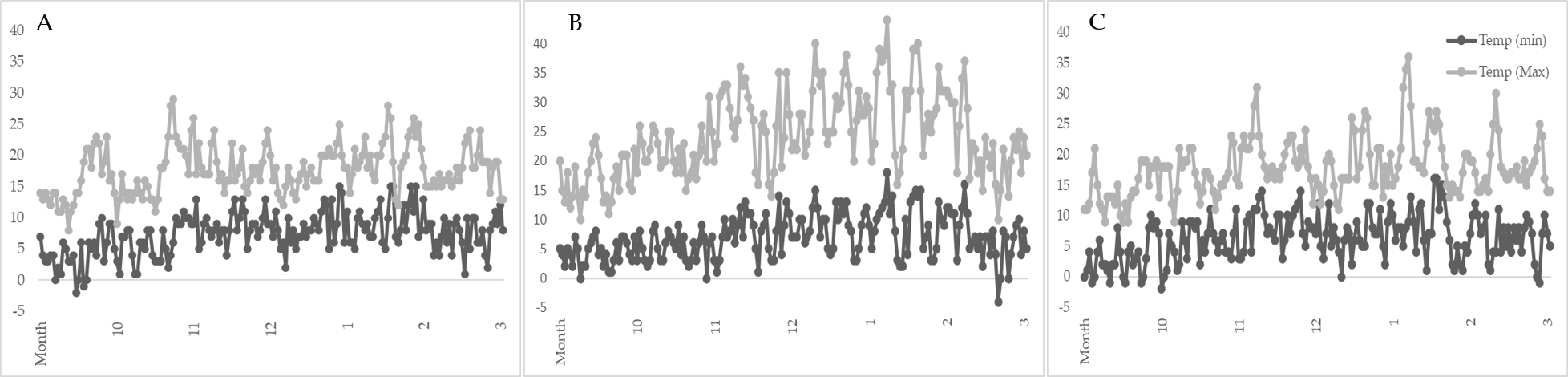

Supplement: Supplementary file 1 [file foods-10-00196-s001.zip › S1. Max_min temp 2016-2019.tif]
